# Error Modeling and Error Control Study of PA/Pine Wood Biomass Composites

**DOI:** 10.3390/polym17141920

**Published:** 2025-07-11

**Authors:** Jiaming Dai, Yanling Guo, Haoyu Zhang

**Affiliations:** Department of Mechanical Engineering, College of Mechanical and Electrical Engineering, Northeast Forestry University, Harbin 150040, China; 18745780386@163.com (J.D.); zhy119803@163.com (H.Z.)

**Keywords:** error prediction model, polymer biomass materials, laser sintering

## Abstract

Laser sintering (LS) technology is one of the most widely commercialized additive manufacturing technologies. However, the popularization of LS technology in civilian applications has long been constrained by accuracy-related issues. Polyamide (PA), as the most mature LS material, still faces challenges in controlling part dimensional errors. Biomass materials, when used as fillers, can improve the printing accuracy of fabricated parts, demonstrating a technically feasible synergy between PA and biomass materials. Therefore, this study analyzes the fundamental material properties of PA/pine biomass composites and investigates error control methods for LS-fabricated parts using PA/biomass materials as feedstock. This study investigates the error modeling of LS-fabricated parts from two perspectives. First, a theoretical mathematical model is established to predict part errors by incorporating material properties, process parameters, and equipment factors. Second, a data-driven model is developed using BP neural network technology based on experimental data to correlate LS process parameters with part dimensional errors. Additionally, the predictive capabilities and compensation effects of both models are examined. The experimental results indicate that the nylon/pine wood biomass composite with a pine wood content of 3 wt% can produce molded parts with a tensile strength of 20 MPa. Additionally, this material exhibits a sintering preheating window range of 10 °C, which facilitates the production of parts with both favorable mechanical properties and dimensional accuracy. Both error prediction models are capable of predicting the dimensional deviations of the parts. The data-driven model demonstrates superior deviation prediction accuracy (approximately 81–91%) for LS parts compared to the theoretical mathematical model (approximately 62–73%). By applying compensation based on the error prediction models, the overall dimensional deviation can be reduced from 1.61–3.49% to 0.41–0.50%. Consequently, the part’s precision grade (according to ISO 2768) is improved from below Grade V to Grade C.

## 1. Introduction

Laser sintering (LS) technology [1,2], as one of the most widely used additive manufacturing technologies today, is increasingly applied in actual production. This has imposed higher requirements on the versatility of consumables, material costs, and part precision [3]. Among numerous materials, biomass composites [4] stand out due to their unique high precision and low-cost advantages. The use of biomass composite materials shows promising potential for fabricating parts with well-controlled dimensional accuracy.

Polyamide (PA) powder [5,6,7], as an important commercial material for laser sintering (LS), has been widely adopted due to its favorable comprehensive properties. However, the high flowability and elevated sintering temperature of PA materials lead to a narrow effective range of sintering process parameters and significant challenges in precision control, posing certain limitations to their development and widespread application. Cai C et al. [8] compared the characteristics of LS- and Multi Jet Fusion (MJF)-fabricated parts using nylon materials. Their study revealed that LS-produced nylon parts exhibit relatively inferior accuracy, with local contour deviations reaching up to 3 mm.

Pine wood powder [9], as a fibrous biomass filler, exhibits low flowability but high decomposition temperature. Zhang H et al. [10] investigated the application of pine wood/polyethersulfone (PES) composites in investment casting technology, demonstrating that pine wood powder contributes to improved dimensional accuracy in fabricated parts. This highlights the complementary properties between pine wood and PA materials. Therefore, biomass composites incorporating PA/pine wood powder are expected to provide a material solution for mitigating dimensional inaccuracies in LS-fabricated parts while retaining adequate mechanical performance.

In practical LS processes, controlling dimensional accuracy remains challenging due to the multitude of factors influencing part precision [11]. This complexity typically necessitates extensive experimental iterations to achieve acceptable tolerances. Conventional error control methods rely heavily on predictive models of LS-induced deviations, which often demand significant temporal and economic investments. Such requirements contribute to the persistently high operational costs associated with LS technology, thereby limiting its flexibility advantages. Recent advances in machine learning techniques [12] offer a promising alternative. By leveraging process test data to establish data-driven predictive models, the efficiency of error prediction can be substantially improved. This approach demonstrates potential for reducing the traditional cost barriers while maintaining prediction reliability.

Malashin et al. [13] proposed a method for predicting the acoustic anisotropy indicators of metal LS parts using deep learning. Based on this deep learning approach, data from other anisotropy constants were utilized to predict Ag, thereby reducing the computational cost of calculating these constants. Tu R et al. [14] compared the effectiveness of Fuzzy Inference Systems (FISs), Artificial Neural Networks (ANNs), and Adaptive Neuro-Fuzzy Inference Systems (ANFISs) for predicting mechanical properties. They established relationships between four parameters—laser power, scanning speed, scanning interval, and part thickness—and the mechanical properties of the parts, achieving effective predictions. Y Wang et al. [15] proposed a deep learning (DL)-based method for the local porosity analysis of laser-sintered Al_2_O_3_ ceramic paste. This approach enables the prediction of part porosity through the laser power parameter. Therefore, data-driven modeling approaches show significant promise for both part error prediction and process parameter compensation. This methodology provides a reliable and efficient technical solution [16] for manufacturing high-precision LS components, addressing the accuracy limitations inherent in conventional processing methods.

The LS process is subject to multiple complex error sources, including slicing layer effects [17], thermal shrinkage [1,18], secondary sintering [19], and optical system errors [20]. These comprehensive error studies make it feasible to establish error prediction models for LS-fabricated parts. For instance, researchers have successfully developed models for predicting sintering defects [21], estimating surface roughness [22], and optimizing scan paths using improved neural networks [23].

In this work, we conduct a comprehensive study focusing on achieving high-precision fabricated components by systematically investigating three critical aspects: (1) the error-inducing effects of material properties and process parameters, (2) the development of error prediction models for manufactured parts, and (3) the implementation of effective error compensation methodologies. A systematic investigation was conducted on the proposed nylon/wood biomass composite material (denoted as PA biomass material) through single-factor and orthogonal tests to evaluate its sintering feasibility and determine the acceptable ranges for both material composition ratios and process parameters. Based on the comprehensive dataset of process parameters and part errors collected in this study, a data-driven model was developed for error prediction. Concurrently, theoretical analysis was performed on the material characteristics of LS-fabricated parts, processing parameters, and equipment factors to establish a simplified theoretical model. A comparative investigation was conducted to evaluate the predictive performance and compensation effectiveness of these two distinct modeling approaches, with the objective of validating their respective capabilities in precision enhancement.

## 2. Experimental Methods and Procedures

### 2.1. Experimental Process and Equipment

**Figure 1:**
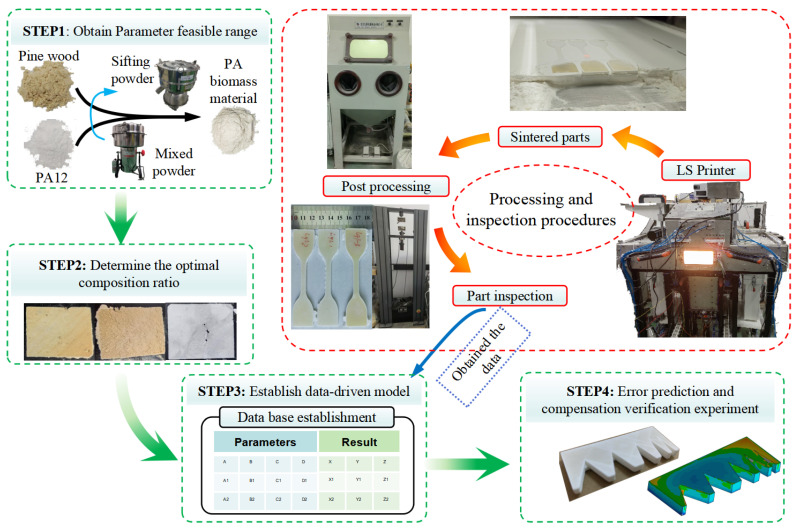
Experimental procedure and research process.

This study focuses on controlling LS part errors through error modeling methods using the PA biomass material. To achieve this, it is necessary to determine the material suitability and acquire valid process parameters and modeling data. Concurrently, this research requires the validation of PA biomass material printability in LS processing combined with a quantitative characterization of their parameter-dependent behavior. Furthermore, an investigation into the influencing factors of the LS printing process is required to obtain the data foundation for establishing a data-driven model of LS part errors. Accordingly, this research is conducted following the four-step methodology illustrated in Figure 1.

Step 1: Select suitable PA powder (provided by Changzhou Xianfeng 3D Technology Co., Ltd., Changzhou, China) and pine wood (provided by Lianyungang Surui Straw Processing Professional Cooperative, Lianyungang, China) powder to prepare effective PA/pine biomass composite materials, determining the maximum reasonable material ratio range.

Step 2: Conduct single-layer LS tests using PA biomass materials to identify feasible composition ratios. Within the viable ratio range, perform basic mechanical property tests using process parameters that yield favorable single-layer forming results. This helps determine an optimal composition ratio, providing a material foundation for subsequent LS parts with improved error control. (The LS equipment utilizes CX_A200 nonmetal LS equipment from Harbin Free Wisdom Technology Development Co., Harbin, China).

Step 3: Perform process parameter experiments using appropriate PA biomass materials to obtain experimental data. These data are used to train a data-driven model while also constructing a theoretical mathematical model based on experimental observations and technical principles.

Step 4: Utilize the established theoretical and data-driven models to conduct error prediction and compensation experiments for LS-fabricated parts.

### 2.2. Experimental Characterization and Testing

The single-layer sintering test parameters for the PA biomass material are detailed in Table 1(1). Initial experiments established the feasible ranges for both process parameters and material composition ratios. Subsequently, building upon the optimal process parameters identified during preliminary testing, we conducted systematic experiments to determine appropriate composition ratios for the PA biomass material. Each test condition was replicated in triplicate, with the experimental parameters documented in Table 1(2). Composition ratios demonstrating superior fundamental mechanical properties were selected for further investigation. Using these optimized materials, we performed orthogonal experiments to evaluate four critical parameters affecting LS-fabricated parts: the scanning interval (A), scanning speed (B), preheating temperature (C), and laser power (D). The complete orthogonal experimental design parameters are presented in Table 1(3).

### 2.3. Error Modeling

For error control in LS-printed parts, the initial step involves establishing error models to facilitate both error prediction and compensation. Here, we analyze the development methodologies and application processes of both theoretical mathematical models and data-driven models.

#### 2.3.1. Theoretical Mathematical Model

**Figure 2:**
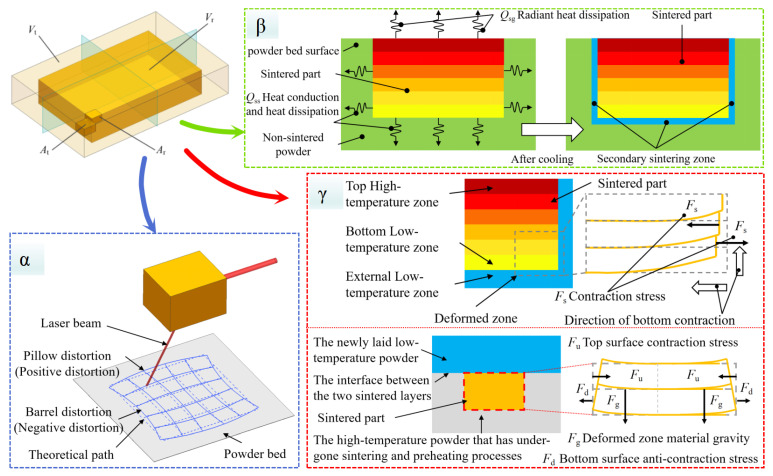
Schematic diagram of discrete division of tiny cells.

The theoretical mathematical model employs principles of differential calculus and finite element analysis, discretizing the part into a finite number of micro-elements. Through the layer-by-layer sintering of these micro-elements, the part achieves interconnectedness to form a complete geometric entity. The resultant dimensional errors are influenced by multiple factors: (1) pre-sintering effects originating from model preparation or material processing; (2) in-process effects accumulating during the sequential sintering of micro-elements; and (3) post-sintering effects developing gradually after fabrication. Figure 2 illustrates the correlation between the three most significant error factors and their corresponding geometric manifestations.

For the geometric volume V of the model, it can be conceptualized as a collection composed of n independent micro-units {A}, where each micro-unit is represented by A(xi,yi,zi). The corresponding variable definitions are presented in Table 1(4). By establishing the spatial relationship between the ideal micro-element and the sintered micro-element, we obtain the displacement vector $\vec{A_{ti}A_{ri}}$ whose magnitude can be expressed by Equation (1).

Through separately representing the vector components along each coordinate axis and mapping the corresponding points to the coordinate components of the ideal unit, we obtain the axial component representation of the displacement vector set $\left\{A_{t\text{→}r}\right\}$, as shown in Equation (2). The displacement functions *F*(x), *G*(x), and *H*(x) for each micro-element can be represented as variables based on geometric transformations of the model, including shrinkage, warpage, and other morphological changes during the sintering process.

(1) $$A_{ti}A_{ri}={\left[X_{ri}-X_{ti},Y_{ri}-Y_{ti},Z_{ri}-Z_{ti}\right]}^{T}$$

(2) $$\left\{\begin{array}{c} {\left\{A_{t\text{→}r}\right\}}_{x}=\left\{X_{\mathrm{ri}}-X_{\mathrm{ti}}\right\}=F(x_{t})\\ {\left\{A_{t\text{→}r}\right\}}_{y}=\left\{Y_{\mathrm{ri}}-Y_{\mathrm{ti}}\right\}=G(y_{t})\\ {\left\{A_{t\text{→}r}\right\}}_{z}=\left\{Z_{\mathrm{ri}}-Z_{\mathrm{ti}}\right\}=H(z_{t}) \end{array}\right.$$

The LS process can generally be divided into three distinct phases: pre-processing, printing formation, and post-processing. Numerous factors contribute to dimensional errors during LS, with varying degrees of influence and different impact stages throughout the printing process. Through practical LS process analysis, we have identified three predominant error sources that significantly affect dimensional accuracy: (1) laser spot position error (denoted as “α”), (2) secondary sintering error (denoted as “β”), and (3) cooling shrinkage error (denoted as “γ”). The mechanisms and effects of these error sources are illustrated in Figure 2.

The laser spot position error (α) primarily originates from optical component imperfections. During laser scanning across the powder bed surface, both pillow distortion (positive distortion) and barrel distortion (negative distortion) occur, directly contributing to part dimensional inaccuracies. The secondary sintering error (β) is predominantly energy-dependent. When sintered material retains temperatures exceeding the preheating temperature after laser energy absorption, adjacent unsintered powder undergoes unintended solidification and adheres to the part’s surface, consequently increasing the final part dimensions. The cooling shrinkage error (γ) is primarily governed by thermal gradients within the material. Significant temperature differentials between the part’s top layer, bottom layer, and sintered regions induce non-uniform shrinkage behavior throughout the component. Furthermore, substantial thermal variations persist even after the part has nominally cooled to ambient temperature. This differential thermal contraction during cooling ultimately leads to a systematic undersizing of the final part’s dimensions.

Given the complexity of error formation mechanisms, we employed polynomial fitting methodology to establish a theoretical mathematical model for predicting LS part dimensional errors. By expanding Equation (2) and retaining the three most significant factors mentioned above, we derived Equation (3), thereby obtaining a theoretically meaningful model with practical physical significance. The laser spot positioning error (α) was calibrated through grid printing tests, yielding a set of deviation constants $\left\{{C0}_{i}\right\}$ for various X-coordinate positions. The secondary sintering deviation (β) was determined through post-sintering measurements, with its effects primarily manifesting at the outermost surfaces of the model. The cooling-induced shrinkage (γ) was estimated based on the material’s overall dimensions, shrinkage coefficient, and temperature variations. The corresponding numerical relationships are expressed in Equation (4).

(3) $$\left\{\begin{array}{l} {\left\{A_{t\text{→}r}\right\}}_{x}=F\left(x_{t}\right)=\alpha_{x}\left(x_{i}\right)+\beta_{x}\left(x_{i}\right)+\gamma_{x}\left(x_{i}\right)+\text{…}\\ {\left\{A_{t\text{→}r}\right\}}_{y}=G(y_{t})=\alpha_{y}\left(y_{i}\right)+\beta_{y}\left(y_{i}\right)+\gamma_{y}\left(y_{i}\right)+\text{…}\\ {\left\{A_{t\text{→}r}\right\}}_{z}=H(z_{t})=\alpha_{z}\left(z_{i}\right)+\beta_{z}\left(z_{i}\right)+\gamma_{z}\left(z_{i}\right)+\text{…} \end{array}\right.$$

(4) $$\left\{\begin{array}{l} \left\{\alpha_{i}\right\}=\left\{{C0}_{i}\right\}\;\\ \beta_{x\text{→}\max}=-\beta_{x\text{→}\min}={C1}_{x}\\ \gamma_{x}=-X\times ∆T\times \lambda \; \end{array}\right.$$

(5) $${∆}_{xi}=F\left(x_{i\_max}\right)-F\left(x_{i\_min}\right)=2\left|F\left(x_{i\_max}\right)\right|=-2X_{i}\times ∆T\times \lambda +2{C0}_{i}+2{C1}_{x}$$

(6) $$L_{xi}=2X_{i}+{∆}_{xi}=2X_{i}-2X_{i}\times ∆T\times \lambda +2{C0}_{i}+2{C1}_{x}$$

By solving Equations (3) and (4), the theoretical mathematical model can be resolved. For instance, the dimensional error in the X-direction can be determined by calculating the difference between the extreme values of its X-direction components, yielding Equation (5). Inputting the corresponding theoretical parameters into this equation enables the derivation of error predictions.

#### 2.3.2. Data-Driven Model

In this work, a data-driven model was established using the Genetic Algorithm Backpropagation (GA-BP) algorithm as an example. Experimental results corresponding to the parameters in Table 1(2),(3) were used to obtain input process parameter data and part error result data. These datasets were employed for basic training through a BP neural network. During the model’s training and testing phases, the final configuration included an imbedded layer count of 10, with data allocation as follows: 60% for training, 20% for testing, and 20% for validation. This approach yielded an effective data-driven model.

**Figure 3:**
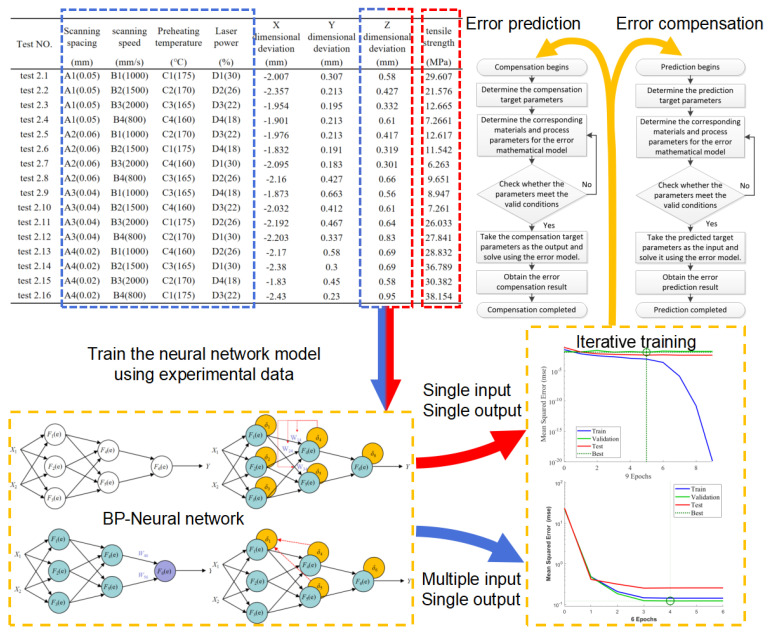
Data-driven model creation process.

Upon acquiring a validated data-driven model, this model can be utilized for part error prediction and compensation, with the corresponding logical relationship shown in Figure 3. Forward-solving the data-driven model yields error prediction results, while setting the error value to zero and performing the inverse-solving of input parameters provides corresponding parameter compensation values.

**Figure 4:**
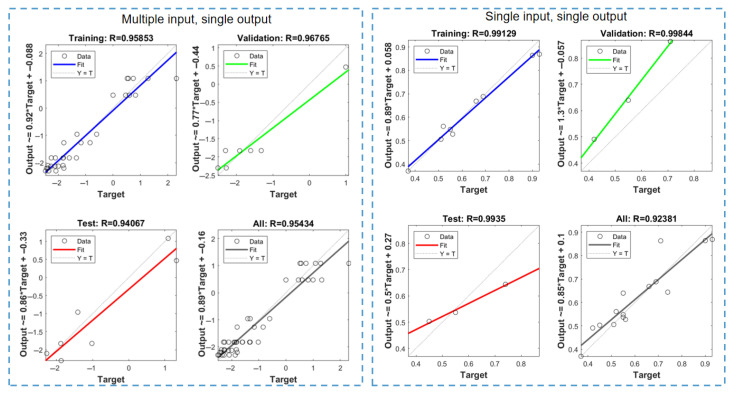
Data-driven model creation process.

During model training and testing, empirical data analysis revealed the successful development of both Single-Input–Single-Output and Multiple-Input–Single-Output data-driven models. The Single-Input–Single-Output model (hereafter referred to as Data-Driven Model 1) effectively correlates the tensile strength of LS-fabricated parts with Z-axis dimensional deformation. The Multiple-Input–Single-Output model (designated as Data-Driven Model 2) establishes the relationship between four key process parameters (as specified in Table 1(3)) and horizontal dimensional deviations in LS components. The training performance characteristics of these neural network-based data-driven models are visually presented in Figure 4.

1. Single-Input–Single-Output Data-Driven Model (referred to as Model 1):

This model effectively correlates the tensile strength of LS parts with Z-direction deformation.

2. Multiple-Input–Single-Output Data-Driven Model (referred to as Model 2):

This model links the four process parameters of LS parts with horizontal dimension deviations.

For Data-Driven Model 1:

The overall regression coefficient exceeds 92.381%, with both the test and validation sets achieving coefficients above 95%. This demonstrates a significant correlation between the strength and Z-direction deformation of LS parts. The test set’s regression coefficient reaches 99.35%, confirming the model’s validity. Additionally, the regression function reveals a contradiction between the goals of high strength and low deformation error in LS parts.

For Data-Driven Model 2:

The overall regression coefficient exceeds 99.129%, with the test and validation sets also surpassing 95%. This indicates a clear correlation between the four process parameters and horizontal dimension deviations of LS parts. The overall regression coefficient result is 95.434%, further validating the model’s effectiveness.

## 3. Results and Discussion

### 3.1. Material Performance Experiments

**Figure 5:**
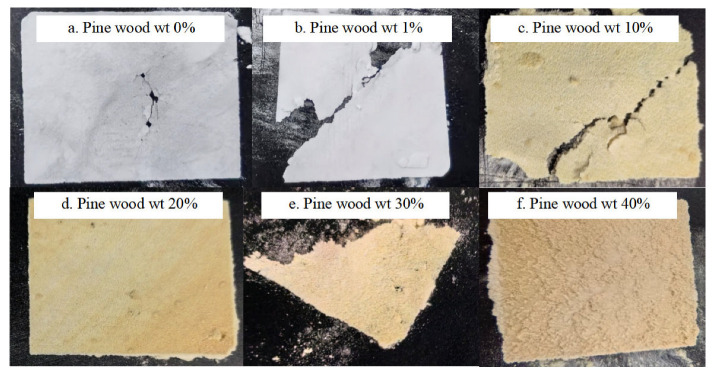
Experimental results of PA/pine wood composite powder component ratio.

Single-layer sintering experiments were conducted using PA biomass material with the process parameters listed in Table 1(2), with the corresponding results shown in Figure 5. Furthermore, composition ratio experiments were performed on the PA biomass material to identify the optimal formulation for error compensation applications.

The experimental results presented in Figure 5 demonstrate that the PA biomass material is suitable for LS processing. A significant influence of pine wood powder content on the single-layer powder spreading process was observed. Notably, when the pine wood content reached 30 wt%, the sintered parts exhibited substantially reduced surface roughness. When the pine wood content exceeds 40 wt%, maintaining adequate powder bed uniformity becomes unachievable during the deposition process. These experimental findings conclusively demonstrate that the PA biomass composite qualifies as a viable LS consumable material, provided the pine wood content is maintained within the 0–40 wt% compositional range.

**Figure 6:**
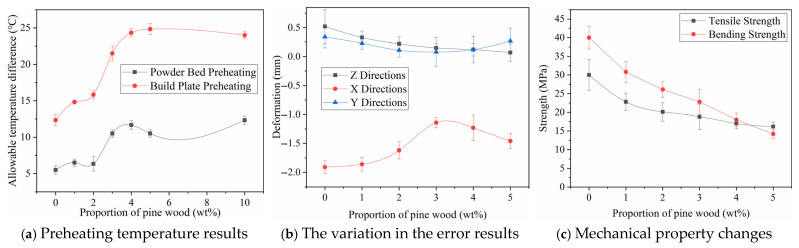
Experimental results of PA/pine wood composite powder component ratio.

By observing Figure 6a, it can be seen that, as the content of pine wood powder increases, the temperature window range of the PA biomass material gradually expands, reaching a peak when the content is near 4%. However, further increasing the pine wood powder content leads to a gradual reduction in the temperature window range. This indicates that the appropriate addition of pine wood powder helps expand the preheating temperature window of LS, improving the thermal adaptability of the PA/pine wood biomass composite. The process parameters employed in these trials are presented in Table 1(3), while Figure 6 summarizes the experimental data, including the temperature requirements, mechanical properties, and dimensional error measurements corresponding to different material compositions.

The reason for this phenomenon is as follows: Pine wood powder has low fluidity and its fluidity is less affected by temperature. PA material has high fluidity, but its viscosity increases significantly as the temperature approaches the glass transition temperature, reducing its fluidity. When the two powders are mixed, the fluidity of the PA biomass material is lower than that of pure PA material at the same temperature, resulting in a larger sintering temperature window. However, an excessively large temperature window increases the temperature gradient during sintering, which further exacerbates part deformation. Therefore, the allowable preheating temperature difference stops increasing after reaching a certain peak.

By observing Figure 6b, it can be seen that as the pine wood powder content increases, the Z-direction deformation of the PA biomass material shows a monotonic decreasing trend. The X- and Y-direction deformation of the PA biomass material first decreases and then increases with increasing pine wood powder content, and the minimum absolute deviation of the part is observed when the pine wood content is approximately 3%. Additionally, it is noted that the absolute deviation in the X-direction is overall greater than that in the Y-direction, and the Y- and Z-direction deviations are positive while the X-direction deviation is negative. This error phenomenon can be well understood by referring to Equation (3). For smaller parts, secondary sintering has a greater impact on part errors, resulting in positive deviations, while for larger parts, thermal shrinkage dominates, leading to negative deviations. The Z-direction size error is mainly due to secondary sintering issues. Although the downward movement in the Z-direction directly affects the part thickness deviation, this movement can be stably controlled within 10 μm by the mechanical structure. For a 2 mm experimental part, the error impact is less than 0.5%, so the influence of the Z-direction motion structure can be ignored.

By observing Figure 6c, it can be seen that as the pine wood powder content increases, the tensile strength and flexural strength of the part show a gradual decreasing trend. Although the decline slows down after the pine wood content exceeds 2 wt%, the good mechanical properties of the PA-based biomass material are its advantage, and lower mechanical properties would significantly limit the material’s applications. Therefore, considering all factors, in this work, the PA/pine wood composite with a pine wood content of 3 wt% is selected as the base material for subsequent research on error control effects.

**Figure 7:**
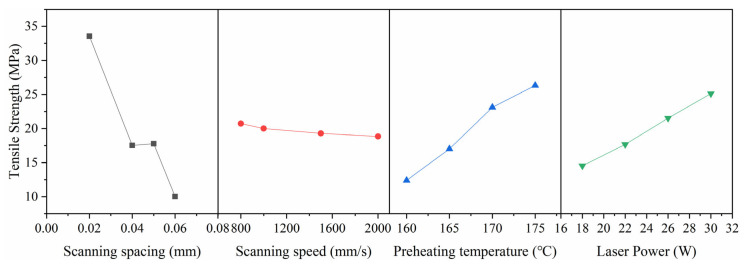

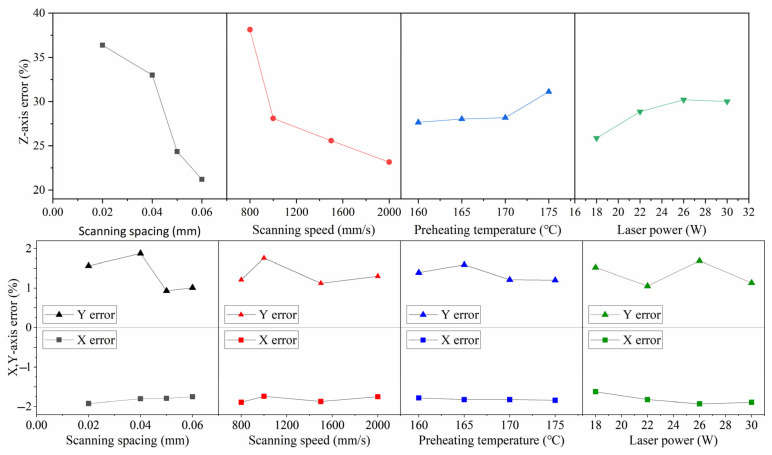
Experimental results.

To systematically investigate the effects of various process parameters on part dimensional accuracy, an orthogonal experimental design was implemented using the parameter combinations specified in Table 1(3). The corresponding measurement results are presented in Table 2, with the observed trends graphically illustrated in Figure 7. The range analysis results for the X- and Z-directions are summarized in Table 3.

The range analysis results for the X- and Z-directions are presented in Table 3. By observing Figure 7, it can be seen that the deviation ratio trend in the Z-direction of the part is consistent with the trend of tensile strength changes. For variations in the four process parameters (corresponding to increased scanning spacing, increased scanning speed, decreased preheating temperature, and reduced laser power), there are cases where both tensile strength and dimensional errors decrease. The trends of these parameter changes also correspond to a decrease in the input power density or energy density [24] of the part. This fully demonstrates the inherent conflict between the goals of achieving good precision and maintaining good mechanical properties. Therefore, in previous research, finding a balance between mechanical property requirements and part error demands has been a key focus in optimizing error control.

Through the range analysis of the experimental results [25], the optimal combination of process parameters can be determined. By analyzing the range results in Table 3, it is found that for the tensile strength of the formed part, the influence of the four parameters is ranked as follows, scanning spacing > preheating temperature > laser power > scanning speed, with the optimal process parameter combination for maximizing strength being A4 (0.02 mm), B4 (800 mm/s), C1 (175 °C), D1 (30 W). For the X-direction error of the formed part, the influence of the four parameters is ranked as follows, laser power > scanning spacing > scanning speed > preheating temperature, with the optimal process parameter combination for minimizing error being A2 (0.06 mm), B1 (1000 mm/s), C4 (160 °C), D4 (18 W). For the Z-direction error of the formed part, the influence of the four parameters is ranked as follows, scanning spacing > scanning speed > laser power > preheating temperature, with the optimal process parameter combination for minimizing error being A2 (0.06 mm), B3 (2000 mm/s), C4 (160 °C), D4 (18 W).

To achieve the dual-objective optimization targeting both minimal dimensional errors and maximal mechanical properties, a mean normalization analytical method was implemented. This approach accounts for the distinct optimization directions required—maximizing tensile strength (assigned a weight of 0.5) while minimizing X- and Z-direction errors (each weighted at −0.25). Table 4 presents the calculated results obtained by applying these weight coefficients to the normalized mean values of parameters Ai, Bi, Ci, and Di. Through a comprehensive analysis of Table 4 data, the optimal parameter combination yielding both peak mechanical performance and minimal dimensional inaccuracies was determined to be A1 (layer thickness: 0.05 mm), B1 (scan speed: 1000 mm/s), C2 (preheating temperature: 170 °C), and D1 (laser power: 30W). This optimized parameter set has been designated as the reference non-compensated LS printing configuration for all subsequent experimental validation studies.

### 3.2. Error Model Experiment

Through the analysis of the basic performance of LS parts and previous studies on the optimization of part error and mechanical property requirements, it is observed that methods for controlling errors often involve sacrificing the material’s mechanical properties. If error models can be further utilized for effective prediction, errors can be compensated based on current technical parameters through the compensation logic shown in Figure 3. Therefore, the aforementioned error models are experimentally analyzed herein.

**Figure 8:**
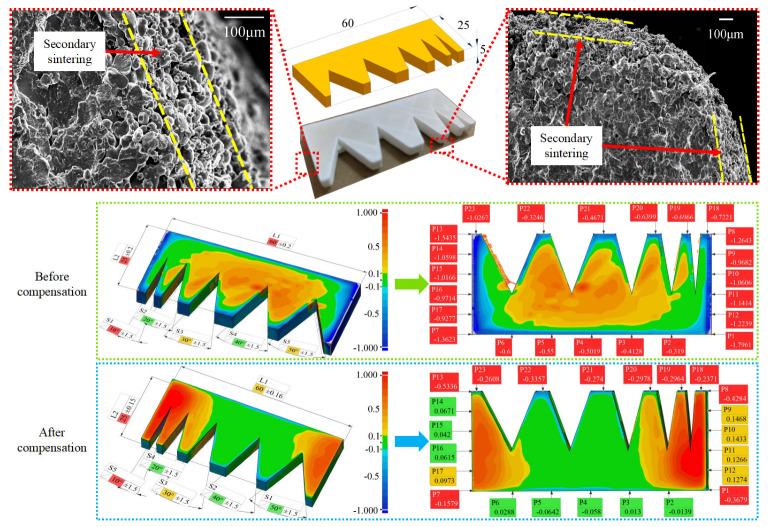
Dimension prediction, detection, and compensation experiment test results for LS parts.

Utilizing the obtained optimal experimental data, this study investigates the efficacy of two models in error prediction and compensation functions. This research focuses on predicting horizontal dimensional errors in PA biomass material LS-fabricated parts, followed by conducting forming experiments and compensation trials. A comprehensive 3D scanning methodology was employed to examine parts both before and after compensation. Figure 8 presents the corresponding part models, SEM observation results, and 3D scanning deviation cloud maps for comparative analysis.

The significant differences observed in the deviation cloud maps of pre- and post-compensation models in Figure 8 primarily result from the alignment process between scanned point clouds and ideal CAD models during 3D scanning inspection. Due to substantial dimensional variations between compensated and uncompensated parts, the software’s automatic alignment reference inevitably introduces certain baseline deviations. To ensure measurement accuracy, we implemented a systematic approach by marking specific length and width dimensions on the parts and collecting data from six uniformly distributed points along each edge. This comprehensive data processing method enables reliable detection results that remain unaffected by potential alignment reference deviations.

As evidenced by the cloud maps in Figure 8, the compensated model exhibits significantly improved continuity in precision-critical regions, clearly demonstrating its superior dimensional accuracy compared to uncompensated parts. For quantitative validation, we processed the comprehensive dimensional dataset by calculating spherical mean values for both nominal dimensions (length/width) and their corresponding deviations, thereby obtaining statistically representative overall dimensional characteristics and deviation metrics.

For directly printed parts, the average overall dimension in the X-direction is 57.9076 mm, with a mean deviation of −2.0924 mm. After compensation using the error model, the average overall dimension in the X-direction for the printed parts is 60.2441 mm, with a mean deviation of 0.2441 mm. For directly printed parts, the average overall dimension in the Y-direction is 24.0345 mm, with a mean deviation of −0.9655 mm. After compensation using the error model, the average overall dimension in the Y-direction for the printed parts is 24.6975 mm, with a mean deviation of −0.302493 mm.

**Figure 9:**
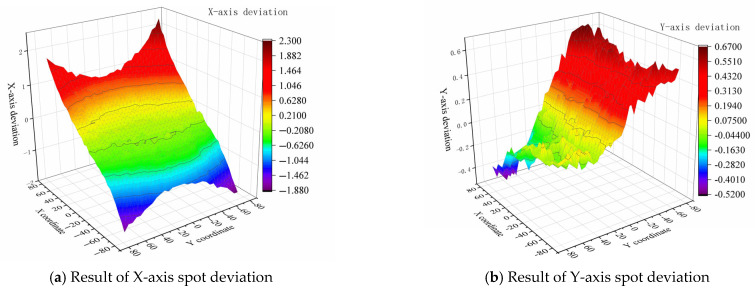
LS equipment laser spot position deviation test results.

The established theoretical mathematical model was employed to estimate part error prediction. In this model, the laser spot position deviation was determined by analyzing grid printing results with a 160 × 160 mm detection area and 5 mm scan spacing. The corresponding laser spot position deviation measurements are presented in Figure 9. Based on actual predicted part dimensions, the α value in the mathematical model was determined by measuring at X = ±30 mm positions, yielding ${C0}_{x=+30}\text{≈}{C0}_{x=-30}\text{≈}0.4$ mm. The secondary sintering thickness, analyzed via scanning electron microscopy (Figure 8), ranged between 0.1 and 0.14mm, leading to a β-term value of ${C1}_{x}=0.12$ mm in the model. For the γ-term, the calculated value incorporated the measured temperature differential 150 °C during printing and the linear expansion coefficient 3.8 × 10^−5^ 1/°C. The complete dimensional prediction for the X-direction is presented in Equation (7).

(7) $$\left\{\begin{array}{l} Lx=2\;\times \;30\;-\;2\;\times \;30\;\times \;150\;\times \;3.8\;\times {\;10}^{-5}\;-\;2\;\times \;0.6+2\;\times \;0.12=58.6980\;\\ Ly\;=\;2\;\times \;12.5\;-\;2\;\times \;12.5\;\times \;150\;\times \;3.8\;\times \;10^{-5}\;-\;2\;\times \;0.4+2\;\times \;0.12=24.2975\; \end{array}\right.$$

The overall X-dimension prediction for the part was obtained through the trained data-driven model shown in Figure 3, which first predicts dimensional deviations and then calculates final dimensions. For a 60 × 25 mm part, the neural network model yielded specific deviation predictions: −0.8477 mm at x = ±30 mm position and −0.529 mm at x = ±12.5 mm position. Based on these symmetrical X-direction predictions, the model’s final dimensional outputs were as follows: X-direction (Lx): 58.3046 mm and Y-direction (Ly): 23.942 mm.

Through the analysis of dimensions and errors before and after compensation, this work has the following three findings:

1. The prediction results of the part’s external dimensions using the theoretical mathematical model fall within ±2% of the actual dimensions, and the prediction error values are within 98.63~98.90% of the actual deviation values. The prediction results of the part’s external dimensions using the data-driven model also fall within ±2% of the actual dimensions, with prediction error values within 99.31~99.62% of the actual deviation values. The error model enables effective dimensional error prediction for parts in both X- and Y-directions.

2. The compensation effect of the error model on the shape errors reduces the overall dimension deviation of the part from 1.61~3.49% to 0.41~0.50%. Corresponding to the actual error results, the absolute X deviation decreases from 2.0924 mm to 0.2441 mm, a reduction ratio of 88.33%; the absolute Y deviation decreases from 0.9655 mm to 0.3025 mm, a reduction ratio of 68.67%. The corresponding ISO 2768 tolerance grade of the part improves from Grade V or lower to Grade C [26]. The error model effectively enhances the precision grade of manufactured components.

3. By comparing the effectiveness of the two error models, it is found that for the theoretical mathematical model, only basic material parameter properties, the laser scanning path testing of the experimental equipment, and a minimal amount of sintering tests are required to obtain an effective theoretical mathematical model. For the data-driven model, a large data foundation is needed, which must be established based on a certain number of experiments to obtain an effective data-driven model. In comparison, the establishment of the theoretical mathematical model is less difficult but has relatively lower effectiveness, while the establishment of the data-driven model is more challenging, but once successfully built, its accuracy is higher than that of the theoretical mathematical model.

## 4. Conclusions

The purpose of this study is to investigate how to achieve LS-printed parts with both good mechanical properties and satisfactory error results. To this end, a complementary PA/pine wood composite material was studied. The LS printing characteristics and the effects of process parameters under different material ratios were analyzed, as well as the impact of various process parameters on the errors and mechanical properties of the parts. Additionally, two error models for LS parts were established: one is a theoretical mathematical model obtained through theoretical analysis, and the other is a data-driven model created based on experimental data. The predictive validity and compensation feasibility of the created error models were further verified using experimental data. The research findings are summarized as follows:

1. The PA/pine wood composite material was determined to have theoretical advantages for achieving high strength and low forming errors. By adopting a weight ratio of 3% PA/pine wood, the composite delivers the best combination of mechanical properties and error results. The optimal process parameters for achieving these two objectives are a scanning spacing of 0.05 mm, scanning speed of 1000 mm/s, preheating temperature of 170 °C, and laser power of 30 W.

2. Through the analysis of factors influencing the LS printing process, a theoretical mathematical model for LS part errors was established. This model considers three significant error factors: laser spot position error “α ”, secondary sintering “β”, and cooling shrinkage “γ”. The model is characterized by low complexity and ease of implementation. The accuracy of the theoretical mathematical model in predicting deviations of LS parts ranges from approximately 62% to 73%.

3. By utilizing LS printing process parameter data, part error result data, and mechanical property result data, a data-driven model for LS part errors was established using BP neural network technology. This model requires a larger data foundation compared to the theoretical mathematical model but achieves more accurate regression prediction results when sufficient data are available. The accuracy of the data-driven model in predicting deviations of LS parts ranges from approximately 81% to 91%.

4. Utilizing the error models for the prediction and compensation of LS parts can help achieve higher-precision LS-printed parts. The compensation method reduces the overall dimension deviation ratio from 1.61%~3.49% to 0.41%~0.50% and improves the corresponding ISO 2768 tolerance grade of the LS parts from Grade V or lower to Grade C.

In summary, leveraging the advantages of the novel nylon/pinewood composite obtained in this study and combining it with the established error model for LS parts can contribute to the efficient and stable production of high-precision LS-printed parts; for LS biomass materials, this approach helps reduce the difficulty of material promotion, expand the scope of material applications, and enhance the applicability of LS technology.

## 5. Challenges and Perspectives

The present study focuses specifically on bio-composite materials with relatively minor deformation. The effectiveness of the proposed error prediction and compensation methods for LS-fabricated parts was evaluated using a limited sample dataset. Future research could expand the sample size or incorporate data from diverse material types to validate the generalizability of the conclusions.

## Figures and Tables

**Table 1:** Material characterization experiments. (1) Parameters of the first orthogonal experiment. (2) Process parameters of the group allocation ratio experiment. (3) Parameters of the second orthogonal experiment. (4) Nomenclature.

(**1**)					
**Test No.**		**Scanning Spacing**	**Scanning Speed**	**Preheating Temperature**	**Laser Power**
		**(mm)**	**(mm/s)**	**(°C)**	**(%)**
test 1.1		A1(0.05)	B1(1000)	C1(175)	D1(30)
test 1.2		A1(0.05)	B2(1500)	C2(170)	D5(24)
test 1.3		A1(0.05)	B3(2000)	C5(167)	D4(18)
test 1.4		A5(0.08)	B1(1000)	C2(170)	D4(18)
test 1.5		A5(0.08)	B2(1500)	C5(167)	D1(30)
test 1.6		A5(0.08)	B3(2000)	C1(175)	D5(24)
test 1.7		A4(0.02)	B1(1000)	C5(167)	D5(24)
test 1.8		A4(0.02)	B2(1500)	C1(175)	D4(18)
test 1.9		A4(0.02)	B3(2000)	C2(170)	D1(30)
(**2**)					
	**Base Plate Temperature**	**Scanning Spacing**	**Scanning Speed**	**Preheating Temperature**	**Laser Power**
unit	°C	mm	mm/s	°C	%
value	165	0.04	1000	173	10
(**3**)					
**Test No.**		**Scanning Spacing**	**Scanning Speed**	**Preheating Temperature**	**Laser Power**
		**(mm)**	**(mm/s)**	**(°C)**	**(%)**
test 2.1		A1(0.05)	B1(1000)	C1(175)	D1(30)
test 2.2		A1(0.05)	B2(1500)	C2(170)	D2(26)
test 2.3		A1(0.05)	B3(2000)	C3(165)	D3(22)
test 2.4		A1(0.05)	B4(800)	C4(160)	D4(18)
test 2.5		A2(0.06)	B1(1000)	C2(170)	D3(22)
test 2.6		A2(0.06)	B2(1500)	C1(175)	D4(18)
test 2.7		A2(0.06)	B3(2000)	C4(160)	D1(30)
test 2.8		A2(0.06)	B4(800)	C3(165)	D2(26)
test 2.9		A3(0.04)	B1(1000)	C3(165)	D4(18)
test 2.10		A3(0.04)	B2(1500)	C4(160)	D3(22)
test 2.11		A3(0.04)	B3(2000)	C1(175)	D2(26)
test 2.12		A3(0.04)	B4(800)	C2(170)	D1(30)
test 2.13		A4(0.02)	B1(1000)	C4(160)	D2(26)
test 2.14		A4(0.02)	B2(1500)	C3(165)	D1(30)
test 2.15		A4(0.02)	B3(2000)	C2(170)	D4(18)
test 2.16		A4(0.02)	B4(800)	C1(175)	D3(22)
(**4**)					
**Symbol**		**Description**			
$A_{ti}$		The i-th theoretical unit.			
$A_{ri}$		The i-th practical unit.			
$\left\{A_{t\to r}\right\}$		The displacement set from theoretical units to practical units.			
$\vec{A_{ti}A_{ri}}$		The displacement vector from the theoretical unit to the practical unit of the i-th element.			
A(xi,yi,zi)		The coordinate representation of the i-th element.			
$X_{ri}$		The practical X-coordinate of the i-th element.			
$X_{ti}$		The theoretical X-coordinate of the i-th element.			
$V_{t}$		Theoretical geometry.			
$V_{r}$		Practical geometry.			

**Table 2:** The results of the orthogonal experiment.

Test NO.	Scanning Spacing	Scanning Speed	Preheating Temperature	Laser Power	X Dimensional Deviation	Y Dimensional Deviation	Z Dimensional Deviation	Tensile Strength
	(mm)	(mm/s)	(°C)	(%)	(mm)	(mm)	(mm)	(MPa)
Test 2.1	A1(0.05)	B1(1000)	C1(175)	D1(30)	−2.007	0.307	0.58	29.607
Test 2.2	A1(0.05)	B2(1500)	C2(170)	D2(26)	−2.357	0.213	0.427	21.576
Test 2.3	A1(0.05)	B3(2000)	C3(165)	D3(22)	−1.954	0.195	0.332	12.665
Test 2.4	A1(0.05)	B4(800)	C4(160)	D4(18)	−1.901	0.213	0.61	7.2661
Test 2.5	A2(0.06)	B1(1000)	C2(170)	D3(22)	−1.976	0.213	0.417	12.617
Test 2.6	A2(0.06)	B2(1500)	C1(175)	D4(18)	−1.832	0.191	0.319	11.542
Test 2.7	A2(0.06)	B3(2000)	C4(160)	D1(30)	−2.095	0.183	0.301	6.263
Test 2.8	A2(0.06)	B4(800)	C3(165)	D2(26)	−2.16	0.427	0.66	9.651
Test 2.9	A3(0.04)	B1(1000)	C3(165)	D4(18)	−1.873	0.663	0.56	8.947
Test 2.10	A3(0.04)	B2(1500)	C4(160)	D3(22)	−2.032	0.412	0.61	7.261
Test 2.11	A3(0.04)	B3(2000)	C1(175)	D2(26)	−2.192	0.467	0.64	26.033
Test 2.12	A3(0.04)	B4(800)	C2(170)	D1(30)	−2.203	0.337	0.83	27.841
Test 2.13	A4(0.02)	B1(1000)	C4(160)	D2(26)	−2.17	0.58	0.69	28.832
Test 2.14	A4(0.02)	B2(1500)	C3(165)	D1(30)	−2.38	0.3	0.69	36.789
Test 2.15	A4(0.02)	B3(2000)	C2(170)	D4(18)	−1.83	0.45	0.58	30.382
Test 2.16	A4(0.02)	B4(800)	C1(175)	D3(22)	−2.43	0.23	0.95	38.154

**Table 3:** Range analysis of parameters in orthogonal experiment.

NO.	A Scanning Spacing	A Norm.	B Scanning Speed	B Norm.	C Preheating Temperature	C Norm.	D Laser Power	D Norm.
	(mm)		(mm/s)		(°C)>		(%)	
TS_K1	(A1)17.8	0.33	(B1)20.0	0.62	(C1)26.3	1.00	(D1)25.2	1.00
TS_K2	(A2)10.0	0.00	(B2)19.3	0.25	(C2)23.1	0.77	(D2)21.5	0.66
TS_K3	(A3)17.5	0.32	(B3)18.8	0.00	(C3)17.0	0.33	(D3)17.7	0.30
TS_K4	(A4)33.5	1.00	(B4)20.7	1.00	(C4)12.4	0.00	(D4)14.5	0.00
Range KR_L	23.5	—	1.91	—	13.9	—	10.6	—
RE_X K5	(A1)2.06	0.21	(B1)2.00	0.00	(C1)2.12	1.00	(D1)2.17	0.86
RE_X K6	(A2)2.02	0.00	(B2)2.15	0.86	(C2)2.09	0.63	(D2)2.22	1.00
RE_X K7	(A3)2.08	0.32	(B3)2.02	0.07	(C3)2.09	0.65	(D3)2.10	0.66
RE_X K8	(A4)2.20	1.00	(B4)2.17	1.00	(C4)2.05	0.00	(D4)1.86	0.00
Range_KR_X	0.187	—	0.167	—	0.065	—	0.361	—
RE_Z K9	(A1)0.487	0.21	(B1)0.562	0.33	(C1)0.622	1.00	(D1)0.600	0.95
RE_Z K10	(A2)0.424	0.00	(B2)0.511	0.16	(C2)0.564	0.16	(D2)0.604	1.00
RE_Z K11	(A3)0.660	0.78	(B3)0.463	0.00	(C3)0.560	0.10	(D3)0.577	0.69
RE_Z K12	(A4)0.728	1.00	(B4)0.762	1.00	(C4)0.553	0.00	(D4)0.517	0.00
Range_KR_Z	0.304	—	0.299	—	0.069	—	0.087	—

**Table 4:** Normalization analysis of dual-objective optimization.

Param. A	Normalized Weight Value	Param. B	Normalized Weight Value	Param. C	Normalized Weight Value	Param. D	Normalized Weight Value
A1	0.06	B1	0.2275	C1	0	D1	0.0475
A2	0	B2	−0.13	C2	0.1875	D2	−0.17
A3	−0.115	B3	−0.0175	C3	−0.0225	D3	−0.1875
A4	0	B4	0	C4	0	D4	0

## Data Availability

The original contributions presented in this study are included in the article. Further inquiries can be directed to the corresponding author.
